# Study of molecular mechanisms of pro-apoptotic activity of NCX 4040, a novel nitric oxide-releasing aspirin, in colon cancer cell lines

**DOI:** 10.1186/1479-5876-5-52

**Published:** 2007-10-30

**Authors:** Anna Tesei, Marco Rosetti, Paola Ulivi, Francesco Fabbri, Laura Medri, Ivan Vannini, Manlio Bolla, Dino Amadori, Wainer Zoli

**Affiliations:** 1 Istituto Scientifico Romagnolo per lo Studio e la Cura dei Tumori, Meldola, Italy; 2 Pathology Unit, Morgagni-Pierantoni Hospital, Forlì, Italy; 3 NicOx SA, Sophia-Antipolis, France

## Abstract

**Background:**

Despite numerous studies aimed at verifying the antitumor activity of nitric oxide-releasing nonsteroidal antiflammatory drugs (NO-NSAIDs), little is known about the molecular targets responsible for their antineoplastic properties. In the present study, we investigated the mechanisms underlying the cytotoxicity of NCX 4040, a novel NO-aspirin with promising antineoplastic action, in *in vitro *human colon cancer models.

**Methods:**

The effect on tumor growth was evaluated in four human colon cancer cell lines (LoVo, LRWZ, WiDr and LoVo Dx) by sulforhodamine B assay, oxidative stress by immunohistochemistry, apoptosis by laddering assay, mitochondrial membrane potential (ΔΨ_m_) by flow cytometry, and apoptosis- and chemoresistance-related markers by western-blot and real-time method, respectively. Prostaglandin E_2 _levels were determined by ELISA.

**Results:**

NCX 4040 produced a higher cytotoxic effect in all the cell lines than that produced by other NO donors tested. In particular, in LoVo and LRWZ cells, NCX 4040 induced a cytocidal effect and apoptosis through p53 and NAG-1 expression, an early ΔΨ_m _collapse, and a sequential release of cytoplasmatic cytochrome c and caspase -9 and -3 active forms. 8-hydroxyguanine lesions, indicative of oxidative stress, were also observed. Conversely, in WiDr line, the drug caused a cytocidal effect, albeit not through apoptosis, and a concomitant increase in COX-2 activity. In LoVo Dx line, characterized by high levels drug resistance and DNA repair-related markers, only a cytostatic effect was observed, again in concomitance with the increase in COX-2 enzyme activity.

**Conclusion:**

This study highlights the multiplicity of mechanisms involved in sensitivity or resistance to NCX 4040 and could provide useful indications for tailored therapy by identifying potentially drug-responsive tumors.

## Background

Nitric oxide-releasing non-steroidal anti-inflammatory drugs (NO-NSAIDS), which consist of a traditional NSAID linked to an -NO_2 _moiety via a molecular spacer, represent a promising class of compounds. These drugs were developed to overcome gastrointestinal and renal toxicities, the main limitations in the long-term use of traditional NSAIDs as chemoprotective agents against colon cancer. The rationale behind NO-NSAID development was based on the protective properties of nitric oxide on the gastric mucosa, similar to that exerted by prostaglandins, whose biosynthesis is inhibited by traditional NSAIDs.

Emerging data indicate that these compounds, in addition to maintaining the chemopreventive properties of traditional NSAIDs, show enhanced safety and efficacy [1-3]. Furthermore, recent studies by our group [4,5] and by other researchers [6] have shown that the NO-aspirin derivative, NCX 4040, inhibits tumor cell growth and induces apoptosis in both *in vitro *and *in vivo *experimental systems. The efficacy of this compound seems to be independent of tumor histotypes [7,8].

For many years the anti-tumorigenic activity of NSAIDs was ascribed to the inhibition of cyclooxygenase (COX) enzymes, in particular, the inducible isoform COX-2. However, in a previous study [4] we showed the inability of NCX 4040 to induce apoptosis in cells with high COX-2 expression, and there is increasing evidence to suggest that NSAIDs may also have a non COX-mediated effect [9]. In the present study we explored other pathways of apoptosis induction and, in particular, investigated the role of the so-called NSAID-activated gene, NAG-1, which belongs to the TGF-β superfamily and is characterized by pro-apoptotic and anti-tumorigenic activities [10,11], in a panel of human colon cancer cell lines.

## Methods

### Cell lines

The study was performed on four cell lines: LoVo and WiDr, obtained from the American Type Culture Collection (Rockville, MD), LRWZ, isolated in our laboratory from a human colon adenocarcinoma, and the multidrug-resistant LoVo Dx, derived from LoVo cells, and kindly provided by Dr. Mario Colombo (Istituto Nazionale Tumori, Milan, Italy). Tumor colon cell lines were maintained as a monolayer at 37°C and subcultured weekly. Culture medium was composed of DMEM/HAM F12 (1:1) supplemented with fetal calf serum (10%), glutamine (2 mM), non-essential aminoacids (1%) (Mascia Brunelli s.p.a., Milan, Italy), and insulin (10 μg/ml) (Sigma Aldrich, Milan, Italy). Cells were used in the exponential growth phase in all the experiments.

### Drugs

**Figure 1:**
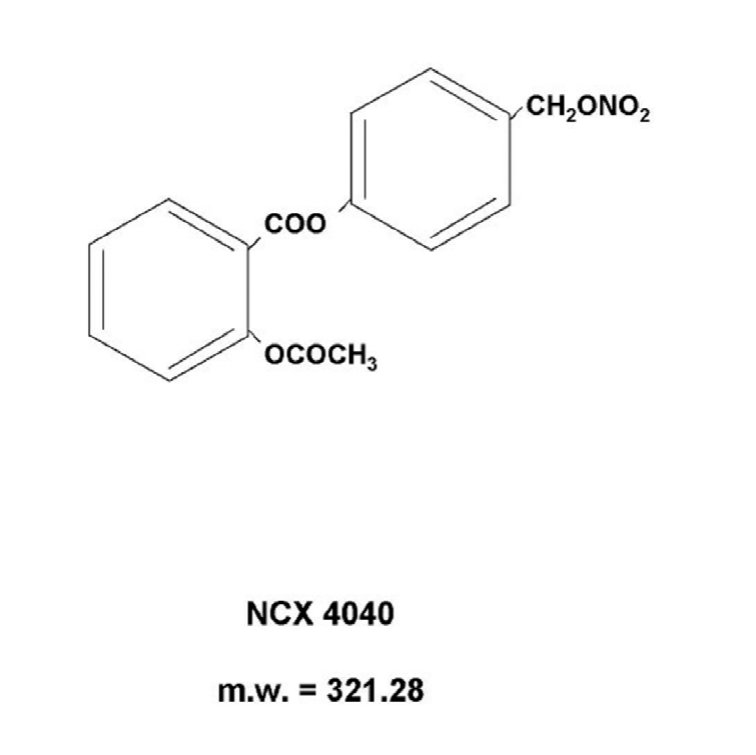
Chemical structure of NCX 4040.

Sodium-nitroprusside dehydrate (SNP), S-nitroso-N-acetylpenicillamine (SNAP), diethylamine-NONOate (NONOATE), NS-398, the selective cyclooxygenase-2 (COX-2) inhibitor (Sigma Aldrich) and NO-aspirin (NCX 4040) (NicOx SA, Sophia Antipolis, France) (Fig. 1) were solubilized in DMSO (Carlo Erba, Milan, Italy) at a concentration of 50 mM, divided into aliquots and stored at -70°C. Drug stocks were freshly diluted in culture medium before each experiment. The final DMSO concentration never exceeded 1% and this condition was used as control in all the experiments.

### In vitro chemosensitivity assay

SRB (sulforhodamine B) assay was used according to Skehan et al.'s method [12]. Briefly, cells were collected by trypsinization, counted and plated at a density of 10,000 cells/well in 96-well flat-bottomed microtiter plates (100 μl of cell suspension per well). Experiments were run in octuplet, and each experiment was repeated three times. The optical density of treated cells was determined at a wavelength of 540 nm using a fluorescence plate reader. In the chemosensitivity assay, NCX 4040 and NO donors were tested at scalar concentrations ranging from 1 to 50 μM for 24 hours followed by a 24-hour drug wash-out.

Growth inhibition and cytocidal effect of drugs were calculated according to the formula reported by Monks [13]: [(OD_treated _- OD_zero_)/(OD_control _- OD_zero_)] × 100%, when OD_treated _was > to OD_zero_. In case OD_treated _was below OD_zero_, cell killing had occurred. The OD_zero _depicts the cell number at the moment of drug addition, the OD_control _reflects the cell number of untreated wells and the OD_treated _reflects the cell number in treated wells on the day of the assay.

### Mitochondrial membrane depolarization analysis

After different exposure times to NCX 4040 tested at a concentration of 10 μM in LoVo and 50 μM in LRWZ lines, cells were harvested, washed once in PBS and then immediately incubated in JC-1 Working solution (BD Biosciences Pharmingen, San Diego, CA) for 10 minutes in a humidified atmosphere at 37°C in the dark. Cells were then washed and resuspended in 1X Assay Buffer (BD Biosciences Pharmingen) and analyzed (FACS Vantage, Becton Dickinson, San Jose, CA). Data acquisition and analysis were performed using CELLQuest software (Becton Dickinson). For each sample, 15,000 events were recorded

### Laddering assay

After different exposure times to NCX 4040 tested at a concentration of 10 μM in LoVo and 50 μM in LRWZ lines, the cells were harvested by trypsinization and their DNA content was isolated using GenElute™ Mammalian Genomic DNA Miniprep Kit (Sigma Aldrich), according to the manufacturer's instructions. The DNA samples were loaded on 1.5 % agarose gel and run at 70 mV for about 1 hour. Internucleosomal DNA fragments were visualized by ethidium bromide under UV light.

### Fluorescence microscopy analysis

Cell-permeable DNA dye 4',6-DAPI and a fluorescence photomicroscope (Zeiss, Axioscope 40) were used to visualize chromatin condensation and/or fragmentation typical of apoptotic cells.

### TUNEL assay

The percentage of apoptotic cells was evaluated by flow cytometric analysis, according to the previously described TUNEL assay procedure [14], in WiDr and LoVo Dx cell lines after exposure to NCX 4040 (10 μM), to the selective COX-2 inhibitor NS 398 (50 μM), or to both drugs. Briefly, after treatment, cells were trypsinized, fixed, exposed to the TUNEL reaction mixture, counterstained with propidium iodide, and then analyzed by FACS.

### Mitochondrial and cytoplasmic fractionations

The ApoAlert Cell Fractionation Kit supplied by BD Bioscience Clontech (Palo Alto, CA) was used according to the manufacturer's instructions, as previously described [8]. Briefly, cells were centrifuged at 600 × *g *for 5 minutes at 4°C and resuspended in 800 μl of fractionation buffer (BD Bioscience Clontech). Cells were passed 20 times through a 22-gauge needle and the homogenates were centrifuged at 700 × *g *for 10 minutes and at 10,000 × *g *for 25 minutes at 4°C.

### Western blot analysis

Cells were treated according to the previously described Western blot procedure [14]. Antibodies used were anti-caspase-3 (polyclonal antibody, Cell Signaling Technology Inc., Beverly, MA, dilution 1:500); anti-caspase-9 (polyclonal antibody, Cell Signaling Technology Inc., dilution 1:500); anti-NAG-1/PTGF-β (polyclonal antibody, Upstate Biotechnology Inc., Lake Placid, NY, dilution 1:500); anti-p53 (PAb 1801, monoclonal antibody, Bioptica, Milan, Italy, dilution 1:400); anti-cytochrome c (ApoAlert Cell Fractionation Kit, BD Bioscience Clontech, dilution 1:100), and anti-COX 4 antibody (BD Bioscience Clontech, dilution 1:500).

The bound antibody was detected by enhanced chemiluminescence (ECL) using an ECL kit (Amersham Pharmacia Biotech, Cologno Monzese, Italy).

### Oxidative damage determination

After a 6-hour exposure of LoVo to 10 μM of NCX 4040 or a 14-hour exposure of LRWZ to 50 μM of NCX 4040, cells were trypsinized and washed in PBS. Approximately 0.5-1 × 10^5 ^cells were plated onto microscope slides using a Cytospin cell preparation system (Shandon, Pittsburgh, PA). Fixed samples, either untreated or treated with NCX 4040, were stained with hematoxylin-eosin and exposed to the antibody that specifically recognizes the presence of 8-hydroxyguanine lesions (anti-8-oxo-dG monoclonal antibody, Trevigen Inc., MD, dilution 1:1000), which are almost exclusively elicited by oxidative stress.

### Prostaglandin E_2 _determination

The determination of prostaglandin E_2 _(PGE_2_) in culture medium was performed using High Sensitivity Prostaglandin E_2 _Enzyme Immunoassay Kit (Assay Designs, Inc., Ann Arbor, MI), according to the manufacturer's instructions.

### mRNA RT-PCR analysis

Total cellular RNA was isolated using RNeasy Minikit (Qiagen, Hilden, Germany). One microgram of RNA was reverse-transcribed into cDNA using iScript (BioRad, Hercules, CA), according to the manufacturer's instructions, and analyzed by real-time RT-PCR (MyiQ System, BioRad) to detect the expression of human breast cancer-resistance protein (BCRP-1), excision repair cross-complementing 1 and 2 (ERCC1-2), topoisomerase I (TOPO I), and thymidylate synthase (TYMS) chemosensitivity markers.

The standard reaction volume was 25 μl and contained 1× SYBR GREEN SuperMix (BioRad), 200 mM of each primer and 2 ul of cDNA template. The mixture was subjected to the following cycling: 95°C for 3 minutes followed by 40 cycles of denaturation at 95°C for 15 seconds, and annealing and extension at 60°C for 30 seconds. Primer sequences were as follows: forward primer, 5'-ACCAACCCTGACGACAGAAGAATC-3' and reverse primer, 5'-GGCGATGTTGAAAGGCACACC-3' for TYMS; forward primer, 5'-AGTCCAAGCATAGCAACAGTGAAC-3' and reverse primer, 5'-CCATCTTTGTGTTTGGTCTTCTCC -3' for TOPO 1; forward primer, 5'-TCAGTCAACAAAACGGACAGTCAG-3' and reverse primer, 5'-TCCTTGGGTTCTTTCCCAGAGC-3' for ERCC1v1; forward primer, 5'-TCATCGCCGCATCAAGAGAAG-3' and reverse primer, 5'-TCATCAGGGTACTTTCAAGAAGGG-3' for ERCC1v2; forward primer, 5'-ATGCTTGGTGGTCTTGTTAAGTGG -3' and reverse primer, 5'-AAGGCTCAGGATCTCAGGATGC-3' for BCRP-1.

The amount of mRNA of each marker was normalized to the endogenous reference β_2_-microglobulin using Gene Expression Macro Software, Version 1.1 (BioRad).

### Statistical analysis

The experimental values of PGE_2 _determination and mRNA RT-PCR analysis represent the median from three independent experiments. Student's t test for paired samples was performed. Differences were considered significant at p values < 0.05 (two-sided).

## Results

### Cell sensitivity to NO-donors and to NCX 4040

**Figure 2:**
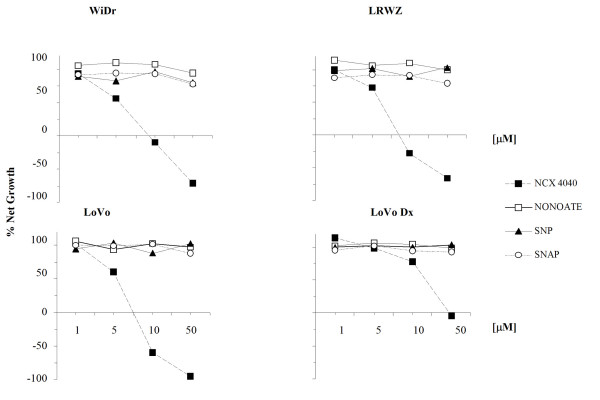
**Activity of different NO donors**. all the cancer cell lines were exposed for 24 hours to NCX 4040 ■, NONOATE □, SNP ▲, SNAP O, at concentrations of 1, 5, 10 and 50 μM, followed by a 24-hour wash-out. Growth inhibition and cytocidal effect of drugs were calculated according to Monks' formula, as reported in Materials and methods. Each point indicates the mean of at least three experiments; SD never exceeded 5%.

The effect of NCX 4040 on cell growth was compared with that of other NO donors such as, NONOATE, SNP, and SNAP. After a 24-hour exposure to different drug concentrations followed by a 24-hour wash-out, NCX 4040 produced both cytostatic and cytocidal effects as a function of the different concentrations tested in three cell lines and only a cytostatic effect in the multidrug-resistant line LoVo Dx, while the other NO donors generated a modest cytostatic effect, failing to reach even IG_50_values (Fig. 2).

### Pro-apoptotic activity of NCX 4040

**Figure 3:**
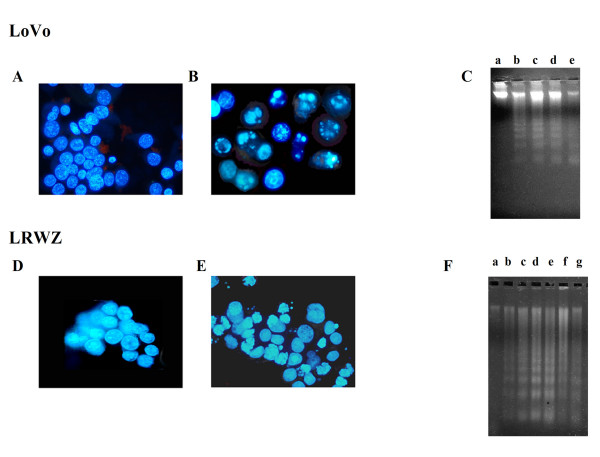
**Fluorescence microscope images (A, B, D and E). Untreated and apoptotic cells in LoVo (A, B) and LRWZ (D,E) cells, respectively, afte**r a 24-hour exposure to 10 μM and 50 μM of NCX 4040, respectively, as evidenced by DAPI staining. **Electrophoresis of genomic DNA samples (C, F)**. Genomic DNA isolated from LoVo (**C**) and LRWZ (**F**) cells after different exposure times to NCX 4040 was electrophoresed on 1.5 % agarose gel to detect internucleosomal DNA fragmentation. (**C**) (10-μM concentration of NCX 4040): lane a, untreated; lane b, 2-hour exposure; lane c, 4-hour exposure; lane d, 6-hour exposure; lane e, 24-hour exposure. (**F**) (50-μM concentration of NCX 4040): lane a, untreated; lane b, 2-hour exposure; lane c, 4-hour exposure; lane d, 8-hour exposure; lane e, 16-hour exposure; lane f, 24-hour exposure; lane g, 48-hour exposure.

As the cytotoxic effect of NCX 4040 was obtained through apoptosis in LoVo and in LRWZ cells but not in the other two lines, we investigated apoptosis mechanisms in LoVo and LRWZ cells at 10- and 50-μM concentrations of NCX 4040, respectively, which represent the minimal drug concentrations capable of causing cell death in these cells lines. Morphological analysis highlighted apoptosis with marked chromatin condensation and/or fragmentation (Fig. 3B-E). Furthermore, the laddering assay showed an induction of apoptosis, which was already detectable after only a 2-hour drug exposure (Fig. 3C-F).

### Apoptosis-related events

**Figure 4:**
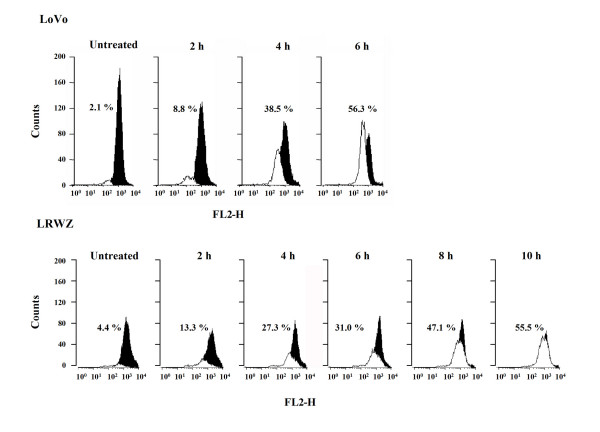
**Analysis of mitochondrial membrane potential (ΔΨ_m_) depolarization**. After different exposure times, variations in ΔΨ_m _induced by 10 μM of NCX 4040 in LoVo and 50 μM in LRWZ were detected using cationic dye JC-1 and flow cytometric analysis. JC-1 exhibits potential-dependent accumulation in mitochondria, as indicated by a fluorescence emission shift from green to red. ΔΨ_m _depolarization is indicated by a decrease in the red/green fluorescence intensity ratio, which is dependent only on the membrane potential and not on other factors such as mitochondrial size, shape or density. FL2-H, median red fluorescence intensity; h, hour.

**Figure 5:**
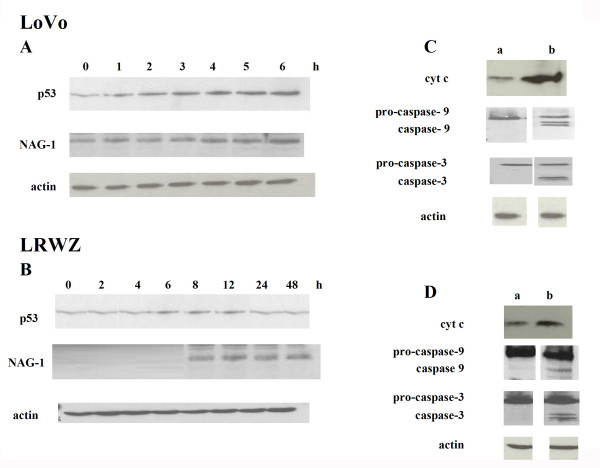
**Protein expression of apoptotic markers**. (**A**) p53 and NAG-1 protein expression in LoVo cells after different exposure times to NCX 4040. (**B**) NAG-1 protein expression in LRWZ cells after different exposure times to NCX 4040. (**C**) Cytochrome c, caspase-9 and -3 protein expression in LoVo cells: lane a, untreated; lane b, 6-hour NCX 4040 exposure (10 μM). (**D**) Cytochrome c, caspase-9 and -3 protein expression in LRWZ cells: lane a, untreated; lane b, 14-hour NCX 4040 exposure (50 μM).

In parallel, the presence of cells with collapse of mitochondrial membrane potential (ΔΨ_m_) was observed in both cell lines. Already present after a 2-hour exposure to NCX 4040, it increased to more than 50% after 6 hours in LoVo and 10 hours in LRWZ cells (Fig. 4). Moreover, in LoVo cells, exposure to NCX 4040 induced an increased expression of both pro-apoptotic protein p53 and NAG-1, starting from a 2-hour and 4-hour drug exposure, respectively, whereas in LRWZ, p53 expression was unaffected and NAG-1 expression was induced after an exposure of 8 hours and was still detectable after 48 hours (Fig. 5A and 5B).

Following pro-apoptotic protein induction and ΔΨ_m _collapse, the release of cytoplasmatic cytochrome c and the presence of caspase-9 and -3 active forms were observed after a 6-hour exposure in LoVo and a 14-hour exposure in LRWZ cells (Fig. 5C and 5D).

**Figure 6:**
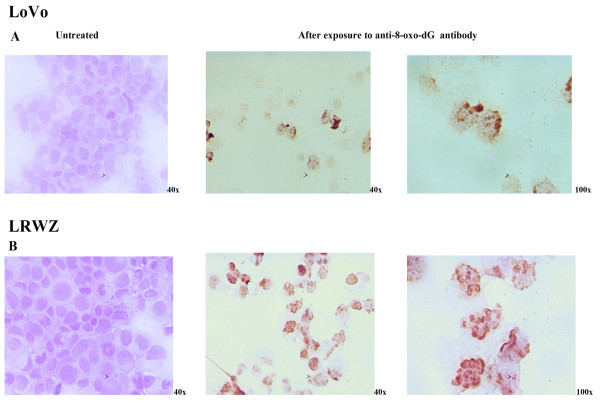
**DNA oxidative damage**. After **(A) **a 6-hour exposure to 10 μM of NCX 4040 in LoVo and **(B) **a 14-hour exposure to 50 μM of NCX 4040 in LRWZ, cells were exposed to the antibody that specifically recognizes the presence of nuclear 8-hydroxyguanine lesions, which are almost exclusively elicited by oxidative stress. Each 100× magnification shows a detail of the corresponding 40× photo. All the pictures are representative of three independent experiments.

Furthermore, the presence of 8-hydroxyguanine lesions in DNA was analyzed as an index of oxidative stress, which is a potential trigger of cell death machinery. A strong nuclear reaction to the antibody that specifically recognizes the oxided nucleotides was observed in apoptotic and non-apoptotic cells in both cell lines (Fig. 6).

### Apoptosis-resistance factors

**Figure 7:**
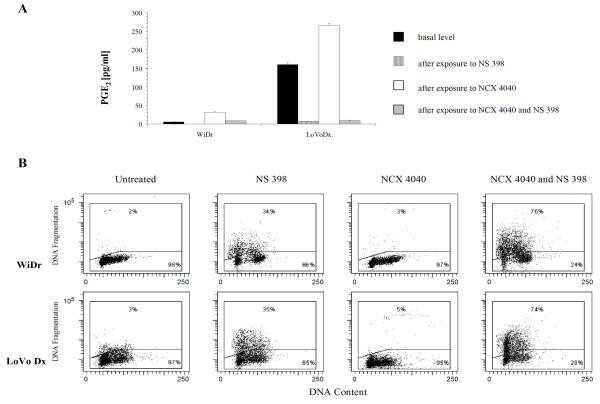
**Quantitative determination of PGE_2 _levels in culture medium by ELISA assay**. **(A)**. Variations induced in WiDr and LoVo Dx cells by a 24-hour exposure to NS 398 (50 μM), NCX 4040 (50 μM), or both drugs. **Apoptosis induction after inhibition of COX-2 enzyme (B)**. Percentage of apoptotic cells after exposure of WiDr and LRWZ cells to NS398, NCX 4040 or both drugs.

For WiDr and LoVo Dx cell lines in which a cytostatic and cytocidal activity or only cytostatic effect was observed, albeit not through apoptosis, we investigated mechanisms potentially responsible for the inability of the drug to induce programed cell death. Among these, the role of COX-2 enzyme and its catalytic activity was evaluated as an expression of PGE_2 _produced by the cyclooxygenase enzyme after exposure to NCX 4040, to the selective COX-2 inhibitor, NS-398, or to both drugs. As expected, NS-398 significantly inhibited PGE_2 _production in both cell lines when used singly or in combination with NCX 4040. Conversely, NCX 4040 induced a significant increase in prostaglandin levels in both LoVo Dx (from 160 pg/ml to 265 pg/ml) and WiDr (from 5 pg/ml to 31 pg/ml) cell lines (Fig. 7A). Moreover, induction of apoptosis (76 % in WiDr, 74 % in LoVo Dx) was observed after inactivation of COX-2 enzyme by NS 398 followed by exposure to NCX 4040 (Fig. 7B).

**Figure 8:**
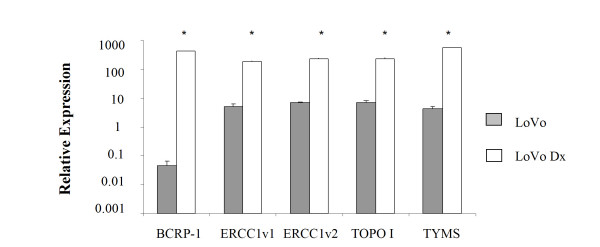
**mRNA expression of chemoresistance- and DNA repair-related markers**. Basal expression of human breast cancer resistance protein (BCRP-1) and DNA repair-related markers (ERCC1-2, TOPO I and TYMS) in LoVo and LoVo Dx cells were analyzed by real-time RT-PCR.

Furthermore, the analysis of chemoresistance- and DNA repair-related markers showed a lower basal expression in LoVo cells than in the corresponding doxorubicin-resistant cell line (Fig. 8). In particular, expression of BCRP-1, TYMS, ERCC1-2, and TOPO I was more than 9000-, 130-, 30- and 30-fold higher, respectively, in LoVo Dx cells than in the LoVo parental line.

## Discussion

NSAIDs, which have long been used for pain relief, fever, and inflammation, are currently under intense investigation to determine the extent and nature of their anti-cancer properties. There is mounting evidence to suggest that the new chemical entities, NO-NSAIDs, show enhanced anticancer activity compared to native NSAIDs. We recently demonstrated that NCX 4040 is highly effective in inhibiting the growth of human colon cancer cells *in vitro *through apoptotic pathways in two of the four cell lines investigated [4]. In the present work, we showed that apoptosis occurs mainly via the mitochondrial pathway, with early mitochondrial membrane depolarization followed by cytochrome c release into the cytoplasm and caspase-9 and -3 activation. This is in agreement with results previously reported by our group for other tumor histotypes [7,8]. Furthermore, the different susceptibility of the various colon cancer cell lines to apoptosis makes this experimental system an interesting model to improve our understanding of the molecular mechanisms that are determinants in triggering cell death.

In the two cell lines prone to apoptosis, we observed the induction of the specific NSAID-activated gene (NAG-1) after a short exposure to NCX 4040, highlighting the role of the aspirin component in the pro-apoptotic activity of the NO-derivative. NAG-1 is a divergent member of the transforming growth factor-β superfamily. Several studies have shown that the anti-tumorigenic and pro-apoptotic role of NAG-1 protein [11,15,16] is regulated by several NSAIDs, such as aspirin, indomethacin and ibuprofen, providing new clues to explain the anti-cancer activities of these anti-inflammatory agents.

The NAG-1 promoter has been characterized and many transcription factors, including p53 [17], are known to regulate this gene. However, the original finding that NAG-1 expression is also induced by NSAIDs in p53 null cell lines [11] seems to indicate the existence of non p53-mediated activation mechanisms. Consistently with this hypothesis, we observed an increment of NAG-1 expression in both LoVo and LRWZ cells and an increment of p53 in only the former cell line.

Our group also hypothesized that the NO component of the molecule plays a pivotal role in the cytotoxic and pro-apoptotic activity of NCX 4040 [4,7,8]. NO is synthesized by normal and neoplastic tissues [18,19] and it has been shown that low endogenous NO levels increase cell proliferation, whereas high levels cause cytotoxicity and apoptosis [20,21]. Moreover, NO released from chemical agents, such as SNP, and other NO-generating molecules has shown anti-proliferative and pro-apoptotic properties in cells from different tumor histotypes *in vitro *and *in vivo *[22-24]. In the present study, we showed that NCX 4040 is more effective in inhibiting cancer cell proliferation than the other three NO-donors, SNP, SNAP and NONOATE. The enhanced effect of NCX 4040 with respect to the classic NO donor compounds is probably largely due to the high -NO release capacity of the spacer component, whose important role in the efficacy of the drug was also highlighted in the work by Kashfi and Rigas [25]. The pivotal role of the -NO molecule in the anti-tumor activity of the drug was further confirmed by the detection, for the first time, of 8-hydroxyguanine lesions, an index of oxidative stress [26], in the DNA of cells that underwent apoptosis after a short exposure to NCX 4040.

The results of the present study also provided some potential explanations for the inability of NCX 4040 to trigger cell death machinery in LoVo Dx and WiDr cells.

In a previous study we showed that COX-2 expression did not change in either WiDr or LoVo Dx cell lines after NCX 4040 exposure [4]. In the present work we analyzed the effect of drugs on the catalytic activity of the COX-2 enzyme, as expressed by PGE_2 _levels. As expected, a considerable inhibition was observed following exposure to NS-398, a specific inhibitor of COX-2 catalytic activity, whereas, surprisingly, a significant increment was seen after exposure to NCX 4040. This finding, already reported by other authors in cancer cell lines with an elevated resistance to apoptosis induced by traditional NSAIDs [27-29] and NO-NSAIDs [30,31], together with the observation that LoVo and LRWZ lines did not express this cyclooxygenase isoform [4], raises intriguing questions about the biological role of COX-2 in the natural history of cancer and also about the mechanisms by which NSAIDs, including NO-NSAIDs, are capable of preventing cancer. Moreover, the onset of apoptosis in cell death-resistant WiDr and LoVo Dx lines after COX-2 inhibition and exposure to NCX 4040 clearly indicates the close correlation between this enzyme and resistance to apoptosis.

In addition to basal elevated COX-2 expression, the high expression of chemoresistance- and DNA repair-related markers in LoVo Dx may explain both the weak cytotoxic effect of NCX 4040 and its failure to trigger apoptosis in this cell line.

## Conclusion

In conclusion, our results indicate that NCX 4040, a novel NO-aspirin, exerts a different cytotoxic activity as a function of the molecular profile of human colon cancer cells. Furthermore, the data we obtained strongly suggest that the NO-releasing moiety (-NO_2_) is responsible for the apoptotic process, which is also enhanced by the aspirin component of the molecule, as shown by its specific induction of the pro-apoptotic protein, NAG-1. In particular, it would seem that the cytocidal effect of NCX 4040 is obtained via mitochondrial pathway-mediated apoptosis in the cells not expressing the COX-2 enzyme, which, conversely, is upregulated after drug exposure in cells failing to undergo apoptosis. These observations, together with the finding that the most resistant cell line was characterized by high levels of drug resistance and DNA repair-related markers, highlight the multiplicity of mechanisms involved in sensitivity or resistance to the drug and could provide useful indications for tailored therapy by identifying potentially drug-responsive tumors.

## Abbreviations

NO-NSAID, nitric oxide-releasing non-steroidal anti-inflammatory drug; SNA, sodium-nitroprusside dehydrate; SNAP, S-nitroso-N-acetylpenicillamine; NONOATE, diethylamine-NONOate; COX-2, cyclooxygenase-2; NCX 4040, NO-aspirin; BCRP-1, breast cancer-resistance protein; ERCC1-2, excision repair cross-complementing 1 and 2; TOPO I, topoisomerase I; TYMS, thymidylate synthase; NAG-1, NSAID-activated gene.

## Competing interests

Manlio Bolla, one of the authors of the paper, is an employee of NicOx SA, the company that produces the compound (NCX 4040) used in the study

## Authors' contributions

AT was responsible for study design, data analysis, and drafting the manuscript. WZ, DA and MB participated in the study design and acted as scientific advisors. AT, MR, PU, FF, IV and LM performed the *in vitro *experiments. All authors read and approved the final manuscript.
